# The Impact of Nutrition Education Intervention with and Without a Mobile Phone Application on Nutrition Knowledge Among Young Endurance Athletes

**DOI:** 10.3390/nu11092249

**Published:** 2019-09-18

**Authors:** Maria Heikkilä, Mikko Lehtovirta, Ossi Autio, Mikael Fogelholm, Raisa Valve

**Affiliations:** 1 Department of Food and Nutrition, Faculty of Agriculture and Forestry, University of Helsinki, P.O. Box 66, 00014 Helsinki, Finland; 2 Institute for Molecular Medicine Finland, University of Helsinki, P.O. Box 20, 00014 Helsinki, Finland; 3 Department of Education, University of Helsinki, P.O. Box 9, 00014 Helsinki, Finland

**Keywords:** sports nutrition, nutrition knowledge, dietary habits, intervention, adolescents, endurance sports, energy intake, carbohydrates

## Abstract

Athletes often have significant gaps in their nutrition knowledge. Thus, the aim of this study was to investigate whether young Finnish endurance athletes’ nutrition knowledge and dietary intake can be improved through an education intervention with or without a mobile food application. Seventy-nine endurance athletes, 18.0 years (SD: 1.4), participated in this randomized, controlled intervention. We compared the effects of participatory nutrition education sessions alone (group EDU) to those including the use of a mobile food application (group EDU + APP) for four days after each session. Both groups attended three 90-min education sessions fortnightly. The participants completed a validated nutrition knowledge questionnaire in Weeks 0, 5, and 17, and a three-day food diary in Weeks 0 and 17. The education plan was based on the Self-Determination Theory and the concept of meaningful learning process. The EDU group’s nutrition knowledge scores were: 78 (week 0), 85 (week 5), and 84 (week 17) and the EDU + APP group’s 78, 86, and 85, respectively. Nutrition knowledge increased significantly (main effect of time (*p* < 0.001)), but we observed no significant group × time interaction (*p* = 0.309). The changes in dietary intakes were minor (*p* > 0.05). The amount of carbohydrates was below endurance athletes’ recommendations throughout the intervention. The reported energy intakes were also below the estimated energy expenditures. In conclusion, nutrition knowledge improved significantly after only three education sessions and food diary feedback, but the mobile app did not improve learning further. However, the nutrition education intervention alone was not enough to change dietary intake.

## 1. Introduction

Training, rest and proper nutrition are the keys for optimal athletic performance. For young athletes, the role of proper nutrition is particularly important because of its role in growth and development [1]. Adequate nutrition knowledge is needed to understand the importance of daily food choices for performance, health and recovery [2,3]. Unfortunately, athletes and coaches often have limited nutrition knowledge [2,4]. Information regarding this knowledge can be used for planning targeted and effective education interventions [5]. By identifying knowledge gaps, an intervention can focus on groups needing more nutrition education, or on specific dietary challenges, such as inadequate carbohydrate consumption [4,6]. To achieve lasting dietary changes, traditional education should be combined with behavioural change strategies and the practical skills needed for following an appropriate diet [7,8,9]. The benefits of prudent dietary changes should be made attractive [7].

Earlier interventions aiming to improve athletes’ nutrition knowledge have varied greatly in their duration and content [9,10,11,12,13,14,15,16]. The education has been provided in either group sessions, mainly lectures [10,15,16] or individual face-to-face sessions [9,11,12]. Athletes’ nutrition knowledge has increased in many of these studies [9,10,11,12,14,15,16]. Other positive outcomes have also been reported, such as increases in self-efficacy, overall number of positive dietary changes and body composition [9,10,16].

While education interventions may require a great deal of time and resources [15], mobile applications (apps) may be helpful in replacing or complementing the use of traditional methods, thus making the interventions more convenient and time-efficient, especially for younger participants preferring new technology [17,18]. In addition to serving as tools for assessing dietary intake in real time, apps may be useful for increasing nutrition knowledge [19]. However, mobile apps have been used for any kind of nutrition promotion, such as improving nutrition knowledge, only for some years and can be regarded as still being in their infancy [19]. Therefore, the best practices for such actions might not be known yet.

The primary aim of this study was to create a new scalable, flexible education programme to improve nutritional knowledge among young Finnish endurance athletes. This target group was chosen because majority of the nutrition knowledge studies have been conducted among team sport athletes far from the Nordic Countries. The randomized, controlled intervention compared the effects of participatory nutrition education sessions alone to those enhanced by a mobile app. We hypothesized that nutrition knowledge would improve in both groups but more among the athletes using the mobile application. In addition to nutrition knowledge, we wanted to study whether any intervention-induced changes occurred in dietary intake.

## 2. Materials and Methods

### 2.1. Participants

We asked endurance sport coaches in two Finnish sports academies, the Finnish Military Sport Federation, and two sports clubs to invite their national- and international-level endurance athletes aged 16–20 to participate in our study. The participants completed an informed consent form before they participated in the study. The study was conducted in accordance with the Declaration of Helsinki, and the study protocol and questionnaires used were reviewed and approved by the University of Helsinki Ethical Review Board (statement 45/2017).

Groups: The participants were randomized into two groups using random permuted blocks, stratified by sex. The EDU group refers to participatory nutrition education sessions. The EDU + APP group refers to participatory nutrition education sessions and the use of the mobile app on a smartphone. The power calculations were based on the results from a prior study on nutrition knowledge [4]. The mean difference in knowledge between athletes and coaches in that study was 8 points (SD: 9), measured using a 79-item questionnaire. Thus, we estimated that the change in the total knowledge score that was possible to achieve and which would benefit the participants, would be approximately eight points. Based on this, we calculated that each group should have 29 athletes (α = 0.01, desired power = 0.80). Due to an estimated drop-out of 25%, 40 athletes were recruited to both groups.

### 2.2. Instruments

Nutrition knowledge questionnaire: We investigated nutrition knowledge using a validated questionnaire for young endurance athletes and coaches [20], which consists of demographic questions and 78 statements (after the deletion of one item that was inappropriate for the target group from the original 79-item questionnaire) in true/false format in sections: (1) nutrition recommendations for endurance athletes, (2) dietary supplements, (3) fluid balance and hydration, (4) energy intake and recovery, and (5) the association between food choices and body image. The content, face, and construct validities both as the test-retest and internal consistency reliabilities were verified during the questionnaire’s development and they were within appropriate limits [20].

Food diary: The athletes recorded everything they ate and drank in a food diary for three successive days (of which one was a weekend day), at baseline and three months after the last education session, to indicate possible long-term changes in the food intakes. The amounts of food were estimated using The Children’s Food Picture Book (2015) developed by the University of Helsinki, Seinäjoki University of Applied Sciences and Folkhälsan Research Center. In this book, three to four different portion sizes of foods and ingredients typical to the Finnish diet were presented. Food diary data were entered and nutritional composition of diet was calculated using AivoDiet dietary software (version 2.2.0.1, Mashie, Malmo, Sweden) which employs the national food composition database Fineli Release 16 (2013).

### 2.3. Procedures

Pilot study: The questionnaire, app, and the structure of the education sessions were tested in a pilot study with eleven 16–22-year old athletes from the Helsinki metropolitan area. They filled in the questionnaire before and after the sessions, which were held on three consecutive days. Minor changes were made after the pilot to keep the schedule of the sessions. After the sessions, we asked the athletes to use the mobile app for a week. Due to their feedback, the food logging period for the actual intervention was shortened to four days.

**Figure 1:**
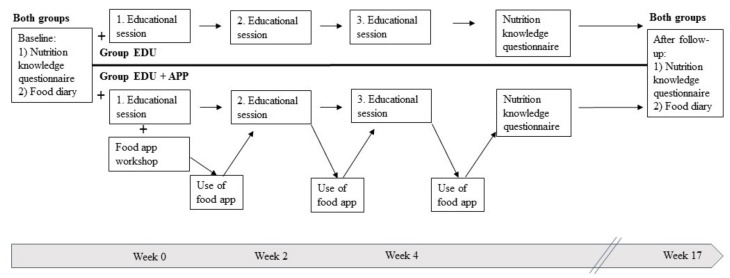
Setting and schedule of the intervention.

Intervention: Figure 1 presents the study timeline. The nutrition knowledge questionnaire was completed before (week 0), after the education intervention (week 5) and after follow-up (week 17). The food diary was completed at baseline and after follow-up. We held three education sessions fortnightly, which were all attended by both groups.

### 2.4. Education Sessions

**Figure 2:**
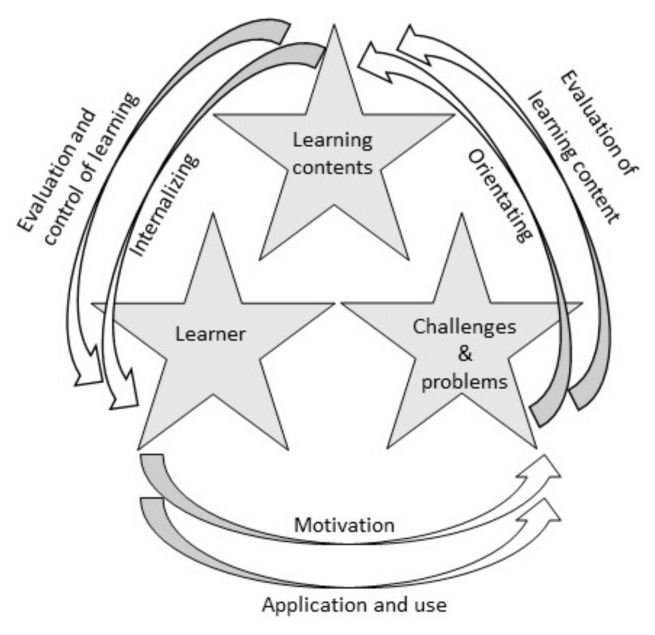
The meaningful learning process. The figure is inspired by Autio [21] and Engeström [22] and is based on Davydov’s concept of developmental teaching [23]. In this intervention, challenges and problems refers to: how to improve the nutrition knowledge among the athletes, learning content to: nutrition knowledge, and learner to: the endurance athletes.

The aim of the education sessions was to increase the nutrition knowledge among the athletes. The education sessions were planned according to the idea of meaningful learning process (Figure 2), explaining the conversion of real-life challenges (e.g., inadequate energy intake) into interesting and motivating educational challenges [21,22,23]. Motivation was the key element of the education plan and student’s learning. Increase in motivation was based on Self-Determination Theory, which is used to explain the role of humans’ inner resources for personality development and behavioural changes [24]. The education sessions aimed to expand the athletes’ feelings of autonomy, competence and relatedness by allowing them to participate in the sessions in the form of discussions, tasks and goal setting, to achieve intrinsic motivation [25]. However, it is impossible to divide the education sessions into parts that addressed only relatedness, only competence or only autonomy. The main idea behind the education sessions was to strengthen all of these feelings.

The education sessions with lectures, discussions, exercises, and individual and group work lasted 90 min each. They were held by a nutritionist (M.H.) and their themes were as follows, based on the findings of the nutrition knowledge study [4] and literature [26].

1. Importance of nutrition for athletic performance, energy requirements, fluids.
2. Carbohydrates, fat, and protein (sources, quality, timing, trends) from the viewpoint of endurance athletes.
3. Certain minerals and vitamins (iron, calcium, magnesium, vitamin D), supplements and challenges (eating on competition days, eating on the road, disordered eating, and weight control).

Feedback on food diaries: The participants received written feedback on their food diaries at baseline and after follow-up from a nutritionist (M.H.). Feedback was an integral part of the education. Energy intake, and macro- and micronutrient intakes from foods and drinks excluding dietary supplements, were shown and compared to recommendations (6–10 g·kg^−1^·day^−1^ or 8–12 g·kg^−1^·day^−1^ carbohydrates for moderate to high intensity endurance training (1–3 h·day^−1^ or >4–5 h·day^−1^), 1.5–2 g·kg^−1^·day^−1^ proteins for high volume of intense training and ~1–2 g·kg^−1^·day^−1^ fat) [27,28]. They also received written feedback highlighting the main strengths and targets for development in their diet, both as a rough estimation of daily total energy expenditure (TEE) and general advice. TEE was calculated using the Harris-Benedict equation for resting energy expenditure (REE) and multiplied by estimated average physical activity level (PAL), 2.1 [29]. Due to the similar training background and after consultation of coaches about athletes’ typical training amounts, the same PAL was used for all athletes in the study.

### 2.5. Mobile App Intervention

The athletes in the EDU + APP group used the photo food journal and nutritional network application MealLogger^®^ with their smartphones for four days after each session. The study used two functions of the app: First, during the four day photographing periods, the athletes were asked to take photos of everything they ate or drank. They were given specific tasks (presented below) to concentrate on when taking photos and/or in additional written descriptions to reinforce their feelings of autonomy and competence. Second, they received written feedback from the nutritionist (M.H.) via the app to support their learning and feelings of relatedness.

Week 1, eating rhythm and fluids: The athletes were asked to concentrate on the number and timing of meals, and the amount of fluids in their diet. They were given feedback twice a week on these points.

Week 2, healthy eating: Feedback was given nearly real-time on the quality of breakfast, lunch and dinner. Before receiving the feedback, the athletes were asked for a self-evaluation of their meals in terms of sources of carbohydrate, fibre, protein, unsaturated fat, and something colourful (vegetables, fruits, berries).

Week 3, variety of food + vitamin D: The athletes were asked to concentrate on the sources of vitamin D in their diet and to log alongside their photographs the food that contained vitamin D. They were encouraged to try new foods and to eat a wide variety of foods. They received feedback on these points twice a week.

### 2.6. Data Analysis

The categorical data are presented as number and percentages. We identified the normality of the variable distributions using Kolmogorov-Smirnov test. For knowledge score or dietary intake variable comparisons between groups and changes in time, we used repeated measures ANOVA (SPSS version 24.0) with age, sex and field of sports as covariates. For macronutrient intakes, we used direct data derived from dietary software instead of Willett’s energy-adjusted intakes [30], as the differences in the values were only minor. We did not exclude potential under-reporters (week 0, *n* = 3; week 17, *n* = 1), because the differences in the analyses were minor. In this study, under-reporting was defined as getting energy from diet less than the estimated REE. All the statistical analyses were conducted using IBM SPSS Statistics for Windows (version 24.0; IBM Corp., Armonk, NY, USA). *p*-values below 0.05 were considered statistically significant.

In the knowledge questionnaire, each wrong answer yielded zero and each correct answer one point. In this paper, “nutrition knowledge score” always refers to the proportion (percentage) of correct answers. The maximum points were 78, in which case the score was 100.

## 3. Results

Of the 79 participants, 62 completed all the nutrition knowledge questionnaires and were thus included in the nutrition knowledge analyses. Sixty-seven completed both food diaries and were thus included in the food intake analyses. Table 1 presents the participants’ background information. Athletes’ mean age was 18.0 years (SD: 1.4). Of the athletes, 56% were male and 44% female. Their main sports were cross-country skiing (*n* = 33) and endurance running/race-walking (*n* = 18). Apart from one athlete, all the participants attended the first lecture. All the athletes completed the first questionnaire, 66 the second, and 67 the third. At baseline, 73 athletes completed the food diary and at the end 67. Of the 42 athletes in the EDU + APP group, 34 used the app (81%). Athletes’ voluntary written (given by 13 athletes) or verbal (given by multiple athletes either individually or in the classrooms during the sessions) feedback on the different parts of the intervention was highly positive, in general.

The mean weight of the athletes in the EDU group was 64.2 kg (SD: 8.8) at baseline and 64.9 kg (SD 8.6) after follow-up. In the EDU + APP group these were 66.4 kg (SD: 8.6) and 67.8 kg (SD: 8.6), respectively. There was a significant increase in weight during the study (main effect of time: *p* = 0.001).

### 3.1. Nutrition Knowledge

**Figure 3:**
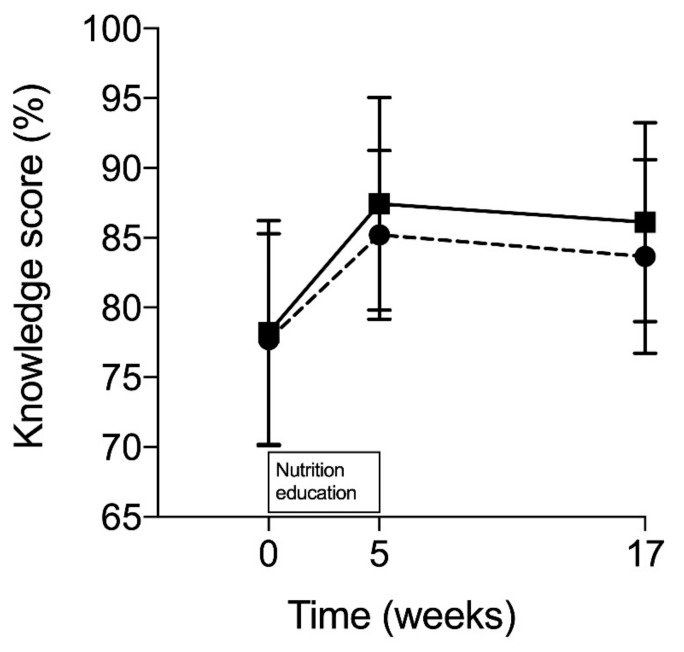
Mean nutrition knowledge scores with SD at baseline, after nutrition education and after follow-up. Black line with squares refers to the EDU + APP group (education sessions + the use of mobile food app) and dotted line with circles to the EDU group (education sessions only). *p* = 0.309 for group × time interaction, *p* < 0.001 for main effect of time, and *p* = 0.309 for main effect of group.

Figure 3 presents the changes in nutrition knowledge. The mean nutrition knowledge scores were 77.7 (SD: 7.6) at their weakest and 87.4 (SD: 7.6) at their best. There was a significant increase in the mean scores during the study (*p* < 0.001). The knowledge scores between the groups did not differ (*p* = 0.309). A comparison of the scores at the end of three months follow-up (week 17) to those at baseline showed that the mean change in knowledge was 6.1 ± 3.7 points in the EDU group and 7.3 ± 6.0 in the EDU + APP group. Immediately after the nutrition education, the mean change in knowledge among the athletes was 6.9 ± 6.2 points in EDU group and 9.3 ± 6.7 in EDU + APP group.

We found a similar tendency in all sections of the questionnaire (Table 2). That is, the athletes in the EDU + APP group scored slightly higher than those in the EDU group, but the differences were not significant (*p* = 0.217 to 0.771). There was a significant increase in the mean scores at the different times of measurement in Section 1, Section 2, Section 3 and Section 4 (*p* < 0.001) but not in Section 5 (*p* = 0.142). The scores were the highest in ‘Fluid balance and hydration’. At baseline, ‘Dietary supplements’ was the most difficult Section.

The male athletes obtained numerically higher mean total knowledge scores (78.3; 87.7; 86.4) than the female athletes (77.6; 85.0; 83.4) but the differences were not significant (*p* = 0.190). The athletes who attended all the lectures achieved higher knowledge scores (77.5; 87.5; 85.3) than those who did not (79.9; 82.2; 84.0) (*p* < 0.001). Although there was no general group difference over the study period in those athletes attending on all lectures or not (*p* = 0.517), there was a significant group × time interaction (*p* < 0.001) and effect of time (*p* < 0.001). A comparison between the athletes in the EDU + APP group showed that the athletes using the app obtained significantly higher knowledge scores compared to the athletes that did not use the app even though they were a part of that group (score 1: 80 vs. 68; score 3: 87 vs. 76) (*p* < 0.001).

### 3.2. Dietary Macronutrient Intake

Although there were some positive changes in the dietary intake, the differences were not significant (*p* > 0.05). The energy intake of the athletes increased but remained under the rough estimations of energy expenditure in both Weeks 0 and 17. While carbohydrate intake increased slightly, it remained below the recommendations (6–10 g·kg^−1^·day^−1^) throughout the study (Table 3).

Fibre intake increased significantly (*p* = 0.001) during the intervention. At baseline, the fibre intake in the EDU group was 32.5 g·day^−1^ and in the EDU + APP group 32.1 g·day^−1^. After the intervention, the intakes were 35.1 and 38.1 g·day^−1^, respectively. The change between the groups was not significant (*p* = 0.181). There were no statistically significant main effects of group × time interaction, time or group in terms of the intakes of sugar and saturated, monounsaturated or polyunsaturated fatty acids. The intakes of sucrose at baseline were 64 g·day^−1^ (group EDU) and 67 g·day^−1^ (group EDU+APP) and after follow-up 66 and 69 g·day^−1^, respectively. The intakes of saturated fatty acids at baseline were 35 g·day^−1^ (group EDU) and 36 g·day^−1^ (group EDU + APP) and after follow-up 33 and 38 g·day^−1^, respectively. The intakes of monounsaturated fatty acids at baseline were 35 g·day^−1^ (group EDU) and 36 g·day^−1^ (group EDU + APP) and after follow-up 33 and 39 g·day^−1^, respectively. The intakes of polyunsaturated fatty acids at baseline were 17 g·day^−1^ (group EDU) and 18 g·day^−1^ (group EDU + APP) and after follow-up 17 and 20 g·day^−1^, respectively.

## 4. Discussion

The main finding of this study was that the nutrition knowledge of the 16–20 year old athletes was significantly improved during a nutrition education intervention consisting of three education sessions and the completion of food diaries, which confirms our first hypothesis. On the contrary to our second hypothesis, the use of the mobile app did not, however, improve the results further.

The age range of 16–20 years was chosen because at that age many of the goal-oriented athletes start their sports high-school or university studies, typically away from their parental home as a consequence of the search of better training opportunities. All of the athletes from the Finnish Military Sport Federation, majority of the athletes of the sports academies and some of the athletes of the sports clubs had already moved from their parental home, as confirmed by the consultation of their coaches. Therefore, most of the athletes of the study did not have parents providing food or cooking food for them on a daily basis. Their training amounts are also on a considerably higher level as compared to child athletes. Therefore, maintaining sufficient energy and nutrient intake is of uttermost importance, also to support young athletes’, some of them still children in the middle of pubertal transition phase, normal growth and development [1].

The nutrition knowledge scores were significantly higher both a week and three months after the sessions compared to the baseline. A higher nutrition knowledge score was notable in all sections, but especially in the questions on dietary supplements. Previous studies have also reported improvements in knowledge [9,10,11,12,14,15,16]. The content and duration of the interventions have varied widely, usually from two to eight months [9,10,11,12,13,14,15,16]. We found that three 90-min participatory nutrition sessions in groups fortnightly was enough to significantly increase young athletes’ nutrition knowledge. In other words, even relatively small use of time and other resources for structured, motivational and science-based nutrition education can promote positive changes in nutrition knowledge which can benefit athletes in their training. We hope that this finding will encourage sport clubs and other sport organizations to increase their nutrition counselling particularly among youth training in the absence of their parents.

The education sessions with lecturing, assignments, goal setting and discussions were designed to increase motivation and the feeling of autonomy, competence and relatedness among the participants, explaining the improvements. The education was based on the Self-Determination Theory and the concept of meaningful learning process [21,24]. The athletes took part in the sessions in the form of discussions, tasks and goal setting, which was intended to increase their intrinsic motivation, as it is a prerequisite for effective learning [25]. Similarly, the personal feedback on the food diaries may have affected the athletes’ intrinsic motivation and led to better engagement in the intervention.

Over 80% of the athletes in the EDU + APP group used the mobile application regularly. However, nutrition knowledge did not improve further among those using of the mobile app. A previous study of athletes using the same app has reported moderate improvements in nutrition knowledge [31]. The app has typically been used for treating patients with disordered eating, weight issues or mental health problems [32]. In general, apps and social media-based methods have been used for dietetic practices [33], such as assessing dietary intake [34] or promoting weight loss [35] but less frequently for improving nutrition knowledge [19]. This study did not use one of the main benefits of mobile apps, namely peer support. This may explain why no significant improvements in knowledge were observed due to the mobile app [19]. Obviously, it should be noted that this study only used one app and the results of this study does not prove that other approaches with other apps or different kind of use of this app could not be beneficial.

As noted previously among Finnish endurance athletes, the questions on dietary supplements and nutrition recommendations appeared the most difficult [4]. Other studies have also reported that the main nutritional misbeliefs among athletes are related to energy density, dietary supplements and proteins [6]. Contrary to our prior results [4], the knowledge of the male and female athletes did not differ significantly. In addition, the age and the main sport of athletes did not affect their nutrition knowledge. However, the age range of the athletes was quite narrow and the energy and nutrient demands both as the training amounts in the different endurance sports were somewhat similar which may explain that these factors did not confound the results.

Regardless of the improved knowledge, the changes in dietary intakes were small, as also noted in other intervention studies [9,36]. The athletes’ energy intakes remained below the rough estimations of energy expenditure throughout the intervention even though they slightly improved. This finding is in line with those of many other studies [9,10,37]. However, potential misreporting and thus under-estimations of energy and nutrient intakes should be considered [18]. TEE was also only an estimation, as we used the same physical activity level for all. The intake of carbohydrates was also inadequate. The carbohydrate intake in this study (4.9–5.4 g·kg^−1^·day^−1^) only meets the recommendations of low-intensity training, and may not be sufficient for young endurance athletes who mostly train twice a day. However, the quality of carbohydrate sources was good as the fibre intake exceeded the Nordic nutrition recommendations (25–35 g·day^−1^) [38]. Fibre intakes considerably below recommendations have also been reported among athletes [9,10]. Male athletes’ higher intakes of energy, carbohydrates, proteins and fats compared to female athletes can be explained by the fact that male athletes need more energy and nutrients due to their higher energy consumption as they are, in general, heavier and taller than female athletes.

The mean knowledge scores were on a satisfactory level already before the intervention thus leaving less room for improvement. In other words, many of the athletes already knew at baseline how to eat. Knowledge is only one of the factors affecting what we eat. Others include psychological, social and economic factors and those related to lifestyle and beliefs, or determinants of food choice [3]. Thus, simply increasing knowledge does not always turn into a certain behaviour if the intention to perform the behaviour is lacking [39]. The prevailing protein hype and restriction of carbohydrates may also have influenced food choices leading to insufficient carbohydrate intakes [40].

The drop-out rate of the participants attending on lectures and completing the questionnaires and food diaries was ~20%. There was no age, sex, nutrition knowledge level or field of sports related factor explaining the drop-outs. Therefore, it was expected that the participants left the study mainly for lack of time or motivation or unsuitable schedules. Also the rate of athletes not using the app in the EDU + APP group was moderate (19%). According to the feedback, many of the athletes liked to use the app and did not feel that it was too laborious or complicated, which reflects the moderate number of athletes not using the app. An interesting finding was that the athletes in the EDU + APP group not using the app obtained significantly lower nutrition knowledge scores compared to the athletes using the app. This may reflect that their motivation regarding the nutrition education may have been lower compared to the other athletes and, therefore, the use of the app seemed like it was too laborious for them.

A strength of this study is that the attendance of lectures and completion of the diaries remained at a high level throughout the study. The athletes were motivated and committed, as reflected by their engagement and in their feedback which was positive with regard to the lectures, personal feedback, and usability of the app. As we succeeded in minimizing the confounding at baseline, the differences between the groups in outcomes at the end were mostly due to differences in education intervention and not to differences in the baseline characteristics. The use of the validated nutrition knowledge questionnaire, especially designed for this study, was also a strength. Finally, the education sessions were planned on the basis of validated psychological theories.

A limitation was that the power calculations were made with the assumption that the knowledge score would increase by eight points. The desired power might have been insufficient for food analyses. Thus, significant changes in food intake may have gone unobserved. However, we only regarded the dietary analyses as exploratory. Second, the personal feedback from food records may have slightly affected food intakes. Therefore, it was not possible to differentiate whether the changes in knowledge were due to education sessions or feedback. However, the feedback was not regarded as a confounding factor but more as an integrated educational method. Third, already at the beginning of the study the diet of the athletes was better than that of the general Finnish population [40]. Due to this, any changes in diet may have been smaller and thus not so visible. Fourth, we did not include any actual control group in our study. Both of the groups of our study did undergo some intervention and, thus, it is not possible to differentiate how much of the change in the knowledge was due to actual learning and how much due to the repetition of the test. However, the main emphasis of this study was to find out whether using the mobile app would further enhance the influence of the educational sessions and, for that purpose, we did have two different study groups. Fifth, self-report bias and underreporting must be considered because the diets were uncontrolled, and they were reported with a food diary.

## 5. Conclusions

Improving athletes’ nutrition knowledge and skills is of great importance to their performance, recovery, overall health, and development. This study used a previously created and validated questionnaire and prior information on athletes’ nutrition knowledge to create an effective education plan aiming to improve athletes’ nutritional skills. By identifying and using information on knowledge gaps, such as dietary supplements and nutrition recommendations for endurance athletes, the intervention was able to focus more specifically on the special needs of athletes.

In conclusion, we observed significant and long-term improvement in nutrition knowledge among athletes after only three education sessions. By using carefully designed education interventions based on validated theories and acknowledging the target population’s needs, nutrition education can be cost effective and lead to considerable improvements in nutrition knowledge. Promoting such education interventions can help to reduce the amount of one-on-one nutritional counselling and save the resources of athletes and sports organizations.

## Figures and Tables

**Table 1:** Background information on athletes presented as numbers and percentages of participants in group.

	Group EDU (*n* = 37)	Group EDU + APP (*n* = 42)
**Sex**		
female	18 (49%)	17 (40%)
male	19 (51%)	25 (60%)
**Main sport**		
cross-country skiing	15 (41%)	18 (43%)
biathlon	5 (14%)	8 (19%)
orienteering	8 (22%)	5 (12%)
endurance running and race-walking	9 (24%)	9 (21%)
triathlon	0 (0%)	2 (5%)
**Attendance of lectures**		
Lecture 1 (Week 0)	36 (97%)	42 (100%)
Lecture 2 (Week 2)	31 (84%)	39 (93%)
Lecture 3 (Week 4)	30 (81%)	29 (69%)
**Completion of questionnaire**		
1st questionnaire (Week 0)	37 (100%)	42 (100%)
2nd questionnaire (Week 5)	31 (84%)	35 (83%)
3rd questionnaire (Week 17)	29 (78%)	38 (90%)
**Completion of food diary**		
1st food diary (Week 0)	33 (89%)	40 (95%)
2nd food diary (Week 17)	30 (81%)	37 (88%)

**Table 2:** Mean nutrition knowledge scores as percentages of correct answers (95% confidence intervals in parenthesis) in the different sections (*n* = 5) of the questionnaire.

	EDU (*n* = 28): Mean Knowledge Score (95% CI)			EDU + APP (*n* = 34): Mean Knowledge Score (95% CI)			*p*-Value (Group × Time Interaction)	*p*-Value (Main Effect of Time)
	Week 0	Week 5	Week 17	Week 0	Week 5	Week 17		
Nutrition recommendations for endurance athletes	75.8	82.8	80.9	75.2	83.6	83.7	0.103	<0.001
	(72.7 to 79.0)	(79.8 to 85.8)	(77.7 to 83.9)	(71.8 to 78.6)	(79.5 to 87.7)	(80.9 to 86.4)		
Dietary supplements	71.4	88.6	82.1	73.5	90.6	90.0	0.276	<0.001
	(62.3 to 80.5)	(81.5 to 95.7)	(75.1 to 89.2)	(67.6 to 79.4)	(86.5 to 94.7)	(85.2 to 94.8)		
Fluid balance and hydration	87.2	91.8	93.4	87.8	94.5	94.1	0.689	<0.001
	(82.6 to 91.9)	(89.2 to 94.5)	(90.0 to 96.7)	(84.2 to 91.4)	(91.4 to 97.7)	(90.5 to 97.7)		
Energy intake and recovery	77.3	86.2	85.7	77.9	88.2	85.3	0.648	<0.001
	(72.6 to 82.0)	(82.4 to 90.0)	(81.8 to 89.6)	(73.3 to 82.6)	(84.2 to 92.2)	(81.4 to 89.2)		
Association between food choices and body image	83.	88.1	86.9	87.9	88.6	90.8	0.345	0.142
	(77.8 to 88.9)	(83.6 to 92.6)	(83.2 to 90.6)	(84.1 to 91.7)	(84.6 to 92.5)	(87.5 to 94.2)		

*p* > 0.05 for main effect of group.

**Table 3:** Energy and macronutrient intakes (95% confidence interval in parenthesis) of athletes.

	EDU (*n* = 30): Mean (95% CI)		EDU + APP (*n* = 37): Mean (95% CI)	
	Week 0	Week 17	Week 0	Week 17
Energy intake (kcal·day^−1^)	2739 (2404 to 3074)	2750 (2434 to 3067)	2931 (2677 to 3184)	3124 (2842 to 3407)
Carbohydrate				
g·day^−1^	320 (278 to 362)	327 (287 to 366)	344 (311 to 378)	368 (327 to 409)
g·kg^−1^·day^−1^	4.9 (4.4 to 5.5)	5.0 (4.5 to 5.6)	5.2 (4.7 to 5.7)	5.4 (4.9 to 6.0)
% of total energy	47 (44 to 49)	48 (46 to 50)	47 (45 to 49)	47 (45 to 48)
Protein				
g·day^−1^	121 (106 to 135)	124 (109 to 139)	135 (121 to 150)	143 (130 to 156)
g·kg^−1^·day^−1^	1.9 (1.7 to 2.0)	1.9 (1.7 to 2.1)	2.0 (1.8 to 2.3) ^‡^	2.1 (1.9 to 2.3) ^‡^
% of total energy	18 (17 to 19)	18 (17 to 19)	18 (17 to 19)	19 (18 to 20)
Fat				
g·day^−1^	98 (83 to 114)	94 (81 to 107)	102 (93 to 112)	108 (98 to 118)
g·kg^-1^·day^-1^	1.5 (1.3 to 1.7)	1.4 (1.3 to 1.6)	1.5 (1.4 to 1.7)	1.6 (1.5 to 1.7)
% of total energy	32 (30 to 34)	31 (29 to 32)	31 (30 to 33)	31 (30 to 33)

‡ *p* = 0.044 for main effect of actually using the application. Male athletes’ average intakes of energy, carbohydrates, proteins and fats were significantly (*p* < 0.05) higher compared to female athletes’ corresponding intakes.
